# Oridonin inhibits epithelial-mesenchymal transition of human nasopharyngeal carcinoma cells by negatively regulating AKT/STAT3 signaling pathway

**DOI:** 10.7150/ijms.48552

**Published:** 2021-01-01

**Authors:** Wei Liu, Gaobo Huang, Yang Yang, Ruixia Gao, Shaoqiang Zhang, Bo Kou

**Affiliations:** 1 Department of Vascular Surgery, The First Affiliated Hospital of Xi'an Jiaotong University, Xi'an, Shaanxi Province 710061, People's Republic of China; 2 National Local Joint Engineering Research Center for Precision Surgery and Regenerative Medicine, The First Affiliated Hospital of Xi'an Jiaotong University, Xi'an, Shaanxi Province 710061, People's Republic of China; 3 Department of Hepatobiliary Surgery, The First Affiliated Hospital of Xi'an Jiaotong University, Xi'an, Shaanxi Province 710061, People's Republic of China; 4 Department of Cadiovascular Surgery, The First Affiliated Hospital of Xi'an Jiaotong University, Xi'an, Shaanxi Province 710061, People's Republic of China; 5 School of Science, Xi'an Jiaotong University, Xi'an, Shaanxi 710061, People's Republic of China; 6 Department of Otorhinolaryngology-Head&Neck Surgery, The First Affiliated Hospital of Xi'an Jiaotong University, Xi'an, Shaanxi Province 710061, People's Republic of China

**Keywords:** oridonin, nasopharyngeal carcinoma, epithelial-mesenchymal transition, AKT, STAT3

## Abstract

Oridonin, derived from *Rabdosia rubescens*, has exhibited anticancer activity in a variety of cancers. However, few studies have explored the effect of oridonin (ORI) on migration, invasion and epithelial-mesenchymal transition (EMT) in nasopharyngeal carcinoma. In our study, the results demonstrated that oridonin significantly inhibited migration and invasion of human nasopharyngeal carcinoma CNE-2Z and HNE-1 cell lines, as depicted by wound healing and Transwell assays. In addition, oridonin increased the expression of E-Cadherin while decreased the expressions of vimentin and twist1 at the mRNA and protein levels in a dose-dependent manner. Interestingly, oridonin also decreased cell mobility in nasopharyngeal carcinoma. The subsequent results of western blotting uncovered that the phosphorylation levels of AKT and signal transducer and activator of transcription 3 (STAT3) were decreased upon oridonin treatment. Furthermore, co-treatment with the AKT activator SC-79 attenuated the anti-metastatic effect of oridonin on nasopharyngeal carcinoma and partially abolished the high expression of E-cadherin and the low expression of twist1 mediated by oridonin. In conclusion, the results revealed that oridonin could repress metastatic phenotype and reverse epithelial-mesenchymal transition (EMT) in nasopharyngeal carcinoma by negatively regulating AKT/STAT3 signaling pathway, suggesting that AKT/STAT3 signaling may be the potential therapeutic target of oridonin against nasopharyngeal carcinoma.

## Introduction

Nasopharyngeal carcinoma is a highly prevalent cancer arise out of the nasopharyngeal mucosal lining, accounting for approximately 12900 new cases worldwide in 2018 1. Owing to the improvement of diagnostic methods and treatment strategies, the 5-year survival rate of nasopharyngeal carcinoma has a prominent increase currently 2. Nevertheless, the high recurrence and metastasis result in a great challenge for the management of this disease 3. Therefore, a better understanding of the mechanism of nasopharyngeal carcinoma metastasis may provide more comprehensive medical evidence for the therapeutic effects. Epithelial-to-mesenchymal transition (EMT) is essential for the initiation of the metastatic cascade 4. During EMT, cells lose the epithelial characteristics, while acquire mesenchymal-appearing properties 5. And cancer metastasis could be driven by the induction of EMT. Hence, it is of great importance to explore the regulated mechanism of EMT.

Oridonin, a bioactive diterpenoid isolated from *Rabdosia rubescens*, is widely used for various pharmacological events in China, including anti-inflammation and anti-tumor 6. Accumulating evidence indicated that oridonin exerted remarkable suppressive activity against colon cancer, gallbladder cancer, breast cancer 7, 8, 9. And oridonin was also found to inhibit EMT in the nude mouse model 10. For nasopharyngeal carcinoma, only one study reported that oridonin induced apoptosis by ROS generation 11. The role of oridonin in nasopharyngeal carcinoma metastasis has not yet been elucidated.

Herein, we focused on exploring the effects of oridonin on metastatic phenotype and epithelial-mesenchymal transition of nasopharyngeal carcinoma CNE-2Z and HNE-1 cell lines. And the regulatory mechanism by which oridonin affected metastatic phenotype would also be validated.

## Materials and methods

### Materials, reagents, and antibodies

Oridonin (C_20_H_28_O_6_), 3-(4,5-dimethylthiazol-2-yl)-2,5-diphenyltetrazolium bromide (MTT) and dimethyl sulphoxide (DMSO) were purchased from Sigma-Aldrich (St. Louis, MO, USA). Antibodies against AKT, phosphorylated-AKT (p-AKT), STAT3, phosphorylated-STAT3 (p-STAT3), E-cadherin, Vimentin, Twist1 and β-actin were obtained from Abcam (Cambridge, UK). Phalloidin dye solution, horseradish peroxidase-conjugated goat anti-rabbit secondary antibody were purchased from Cell Signaling Technology, Inc (Beverly, MA, USA).

### Cell lines and cell culture

Human nasopharyngeal carcinoma cell lines CNE-2Z and HNE-1, and nasopharyngeal epithelial cell NP460 were purchased from American Type Culture Collection (Manassas, VA, USA). These cell lines were cultured in RPMI 1640 medium containing with 10% fetal bovine serum (Gibco, Grand Island, NY, USA), 100 U/ml penicillin and 100 μg/ml streptomycin. And the cells were seeded at 37 °C in a humidified atmosphere with 5% CO_2_.

### MTT assay

A modified MTT assay was used to detect the cell cytocoxicity. In brief, 0.8 × 10^4^ CNE-2Z and HNE-1 cells were plated per well in 96-well plates and incubated with RPMI 1640 medium at a volume of 200 μl. Then cells were treated with the increasing concentrations of oridonin. After incubation for 24 h, 20 μl of MTT dye solution (5.0 mg ml^-1^) was added to each well with 180 μl medium for another 4 h. Subsequently, cells were lysed with dimethyl sulfoxide (DMSO) to dissolve the violet blue crystals. The optical density (OD) of each well was quantified at 490 nm wavelength on microplate reader (Bio-Rad, Hercules, CA, USA). The growth inhibitory rate was calculated as: [(OD 490_control cells_-OD 490_treated cells_)/ OD 490_control cells_] ×100.

### Wound healing assay

Nasopharyngeal carcinoma CNE-2Z and HNE-1 cells were plated onto six-well plates. After cell density reached into 90 % confluence, the wounds were scratched across the monolayer with the tip of a 200-µl pipette. After cultured in a serum-free medium with oridonin treatment for 24 h, five fields (100 ×) were randomly captured from each scratch wound and visualized by microscope to evaluate the migratory capacity.

### Transwell migration assay

It was performed using human nasopharyngeal carcinoma CNE-2Z and HNE-1 cells upon oridonin treatment. 6 × 10^4^ cells with 200 μl serum-free medium were added into the upper chamber, while 800 μl of 10% FBS-supplemented medium were then plated onto the lower chamber. After incubation of 24 h, the cells that migrated to the lower surface of the filter were fixed in 4 % paraformaldehyde and stained with 0.1% crystal violet for 10 min. Cells in five random fields were counted and visualized using an inverted microscope at 100 × magnification.

### Matrigel invasion assay

It was conducted to explore the impact of oridonin on the invasion of nasopharyngeal carcinoma with a Millicell chamber (Millipore, Billerica, MA, USA). The membrane with 8 μm pore size in the upper chamber was pre-coated with 50 μl Matrigel (Matrigel: serum-free medium 1:5). CNE-2Z (12 × 10^4^) and HNE-1 (10 × 10^4^) cells were prepared, and the rest of the steps was performed following the instructions of Transwell migration assay.

### Quantitative real-time PCR assay

CNE-2Z and HNE-1 cells were pre-treated with certain concentrations of oridonin and the total RNA were extracted by TRIzol reagent (Invitrogen, Carlsbad, CA, USA). Then the RNA was reversely transcribed to complementary DNA (cDNA) using a PrimerScript RT reagent Kit (Takara, Dalian, China). Subsequently, the quantitative real-time PCR was carried out by SYBR Green Master Mix. The sequences of primers for PCR amplification were forward 5'-CGAGAGCTACACGTTCACGG-3' and reverse 5'-GGGTGTCGAGGGAAAAATAGG-3' for E-Cadherin (119 bp); forward 5'-GACGCCATCAACACCGAGTT-3' and reverse 5'-CTTTGTCGTTGGTTAGCTGGT-3' for Vimentin (238 bp); forward 5'-GTCCGCAGTCTTACGAGGAG-3' and reverse 5'-GCTTGAGGGTCTGAATCTTGCT-3' for Twist1 (156 bp); forward 5'-CATGTACGTTGCTATCCAGGC-3' and reverse 5'-CTCCTTAATGTCACGCACGAT-3' for β-actin (250 bp). β-actin was used as an internal reference.

### Western blotting

Briefly, nasopharyngeal carcinoma CNZ-2Z and HNE-1 cells were collected upon oridonin treatment, and the cell lysates were extracted with protein lysis buffter. After centrifugation and denaturation, the extracts containing about 30-60 µg were subjected to SDS-PAGE (10 % or 15 % gels) and transferred onto polyvinylidene fluoride membranes (Millipore, Bedford, MA, USA). Membranes were blocked with 5% non-fat milk for 1 h and then probed with primary antibodies against E-cadherin, Vimentin, Twist1, AKT, p-AKT, STAT3, p-STAT3 and β-actin overnight at 4 °C. Subsequently, the membranes were washed with TBST buffer and incubated with horseradish peroxidase (HRP)-conjugated IgG antibody for 1 h at room temperature. Finally, the blots were detected using ECL Substrate and exposed to X-ray film according to the manufacturer's protocol.

### Immunofluorescence microscopy

The immunofluorescence staining of F-actin was used to detect the cell mobility. Briefly, cells on slides were washed with PBS buffer and fixed with 4% paraformaldehyde for 15 min. Then cells were washed again and permeabilized with 0.1% Triton X-100 for another 5 min. Subsequently, cells were stained by Phalloidin dye solution (100 μg ml^-1^) and DAPI dye. The images were captured by Nikon (Tokyo, Japan) A1 confocal microscope.

### Plasmid transfection

JAK2 cDNA was cloned into pcDNA3.1 vector. After nasopharyngeal carcinoma CNE-2Z or HNE-1 cells reached 80 % confluency for plasmid transfection, the cells were transiently transfected with X-treme GENE HP DNA Transfection Reagent (Roche, Germany) for certain time following manufacturer's instructions, and used for the subsequent experiments.

### Statistical analysis

Data were represented as mean ± standard deviation and analyzed using GraphPad Prism (vesion 6.0) software. Differences between two groups were assessed by Student's *t*-test (two-sided). *P* values less than 0.05 was considered statistically significant.

## Results

### Oridonin exerts anti-proliferation effect in nasopharyngeal carcinoma CNE-2Z and HNE-1 cells

**Figure 1:**
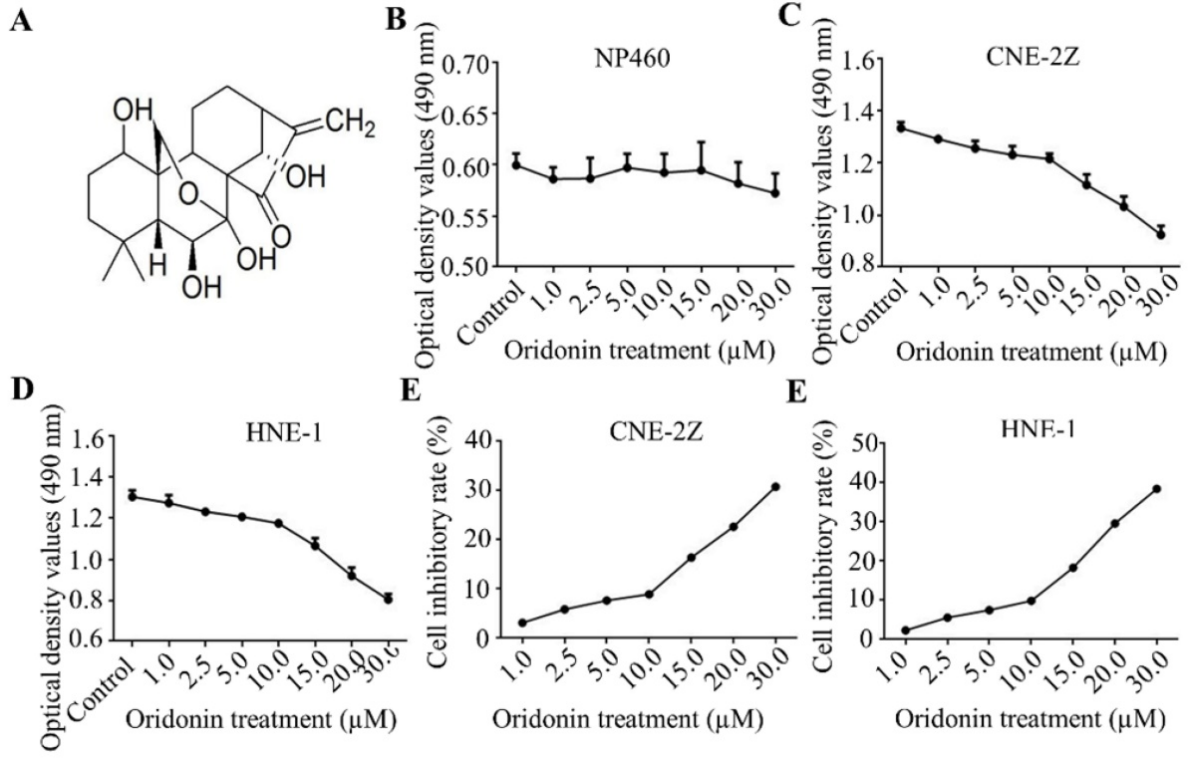
Oridonin inhibited the growth of human nasopharyngeal carcinoma cells. A. The chemical structure of oridonin. B. The cytotoxic effect of oridonin on nasopharyngeal epithelial cell NP460. The optical density of oridonin in NP460 cells was shown as mean ± SD. C-F. The cytotoxic effect of oridonin on nasopharyngeal carcinoma CNE-2Z and HNE-1. Cells with 90 % density were exposed to various concentrations of oridonin (1, 2.5, 5, 10, 15, 20, 30 μM). The viability of these two cell lines were evaluated by a modified MTT assay. The optical density and cell inhibitory rate of oridonin in CNE-2Z (B and D) and HNE-1 (C and E) cells were shown as mean ± SD.

Firstly, Fig. 1A exhibited the chemical structure of oridonin with the molecular structural formula C_20_H_28_O_6_ and a molecular weight of 364.44 g/mol. The results of MTT assays showed that oridonin had no prominent cytotoxic effect on nasopharyngeal epithelial cell, while the growth of nasopharyngeal cacricnoma CNE-2Z and HNE-1 cells was remarkably inhibited by oridonin at a concentration of ≥ 10.0 μM (Fig. 1B-1F). In view of the above, oridonin at 10 μM (a < 10% inhibitory rate) was considered as the adequate concentration in the next studies, to exclude the suppressing interference from nasopharyngeal carcinoma proliferation by oridonin.

### Oridonin inhibited the migration and invasion of human nasopharyngeal carcinoma cells

**Figure 2:**
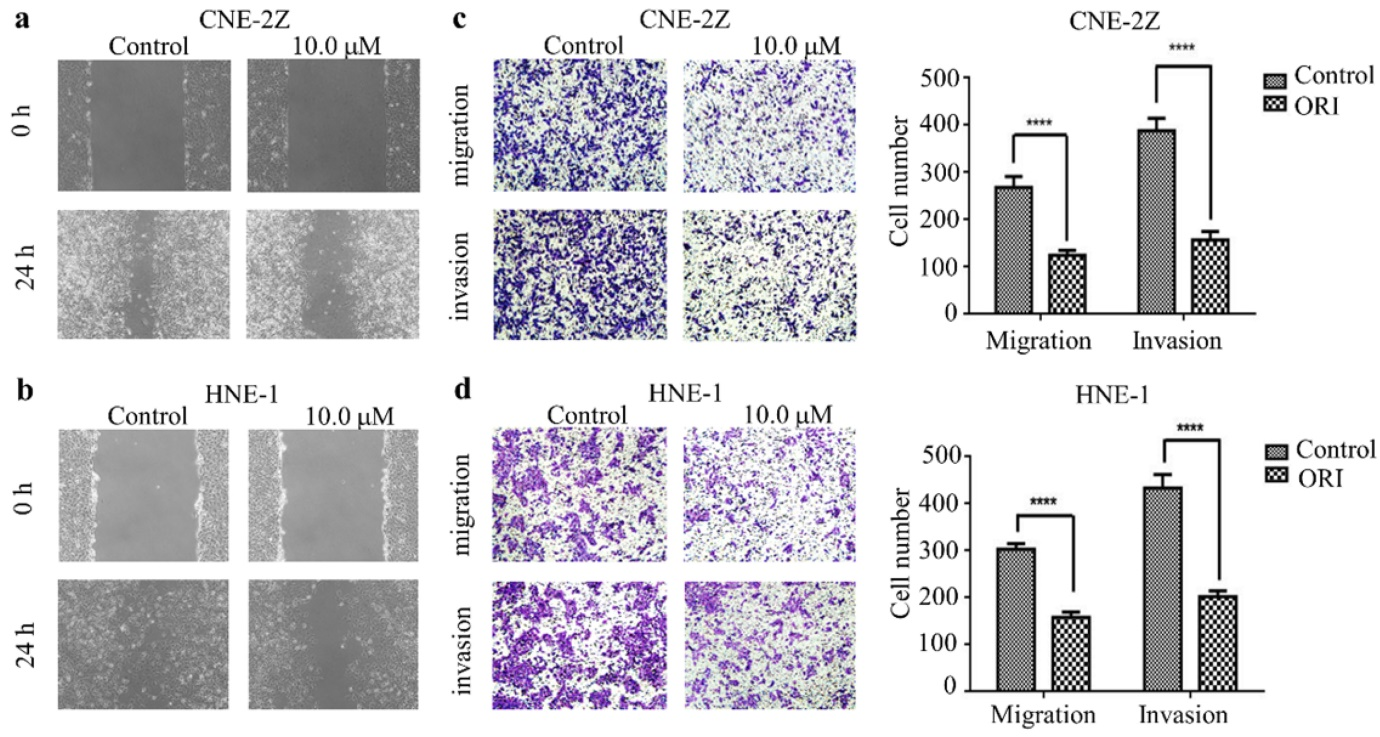
Oridonin (ORI) inhibited the migratory and invasive activity of nasopharyngeal carcinoma CNE-2Z and HNE-1 cells. The width of scratches was measured upon oridonin treatment or not at 0 and 24 h in nasopharyngeal carcinoma CNE-2Z (A) and HNE-1 (B) cells. And using Transwell migration assay and Matrigel invasion assay, the number of migrated or invaded cells (C and D) treated with oridonin per chamber was counted (magnification, ×100). And quantification of three independent experiments was shown (****P<0.0001).

To identify the function of oridonin on migratory and invasive characteristic in nasopharyngeal carcinoma cells, wound healing assay was conducted by a microscope at 0 and 24 h. The results demonstrated that the wounded areas was wider in the group of oridonin treatment than that in control group in nasopharyngeal carcinoma CNE-2Z and HNE-1 cell lines (Fig. 2A and 2B). Additionally, oridonin in the CNE-2Z cells could weaken the migratory ability after 24 h (Fig. 2C). Likewise, oridonin resulted in a dramatical decrease in migration in HNE-1 cells compared to the control group (Fig. 2D). These data indicated that oridonin had the ability to suppress migration in the human nasopharyngeal carcinoma CNE-2Z and HNE-1 cells. Subsequently, Matrigel invasion assay was performed to explore whether oridonin could affect the invasiveness of nasopharyngeal carcinoma cells. The findings showed that oridonin effectively decreased invasive capability of CNE-2Z and HNE-1 cells (Fig. 2C and 2D).

In summary, the results suggested that oridonin exhibited a prominent anti-metastatic ability in human nasopharyngeal carcinoma cells, as evidenced by the results in the wound-healing and Transwell assays.

### Oridonin reversed EMT in human nasopharyngeal carcinoma cells

**Figure 3:**
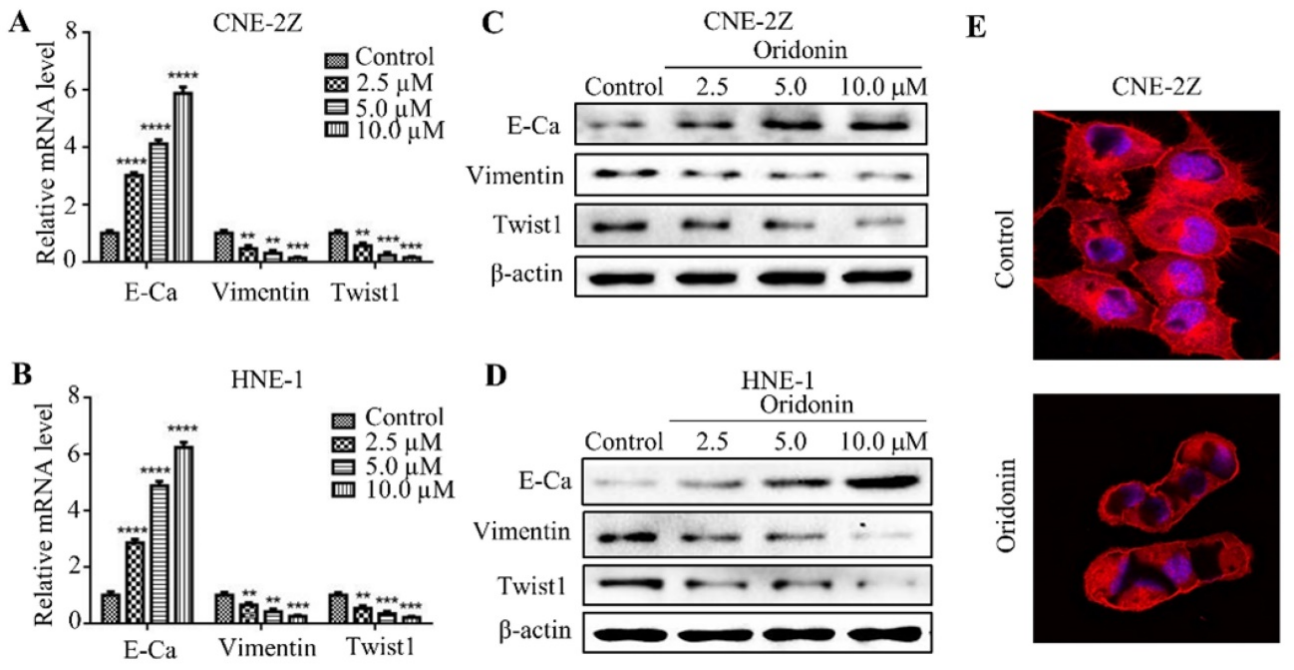
Oridonin reversed epithelial-mesenchymal transition in nasopharyngeal carcinoma cells. Quantitative real-time PCR was conducted to examine the expression of E-cadherin (E-Ca), vimentin, and twist1 in nasopharyngeal carcinoma CNE-2Z (A) and HNE-1 (B) cell lines (**P*<* 0.01, ***P<0.001, ****P<0.0001). Western blotting was used to detect the protein levels of E-cadherin, vimentin, twist1 and β-actin in nasopharyngeal carcinoma CNE-2Z (C) and HNE-1 (D) cells treated with various doses of oridonin. Representative blots from three experiments were presented. (E) F-actin staining with immunofluorescence microscopy was performed to validate the mobility capacity of CNE-2Z cells upon oridonin treatment (10 μM).

EMT is closely correlated with tumorigenesis and cancer progression. Therefore, we detected the effect of oridonin on mRNA and protein levels of EMT in nasopharyngeal carcinoma cells. As expected, the data uncovered that the expression of E-cadherin increased, while the expressions of vimentin and twist1 decreased under oridonin treatment in a dose-dependent manner (Fig. 3A-3D), indicating that oridonin could reverse EMT. Then confocal immunofluorescence microscopy was used to assess the stress fibers (F-actin) in nasopharyngeal carcinoma CNE-2Z cells upon oridonin treatment or not. Unsurprisingly, oridonin was found to decrease the bundled F-actin (stress fibers), indicating that oridoing inhibited the mobility of CNE-2Z cells (Fig. 3E).

### Oridonin downregulated the expression of p-AKT and p-STAT3 in nasopharyngeal carcinoma cells

**Figure 4:**
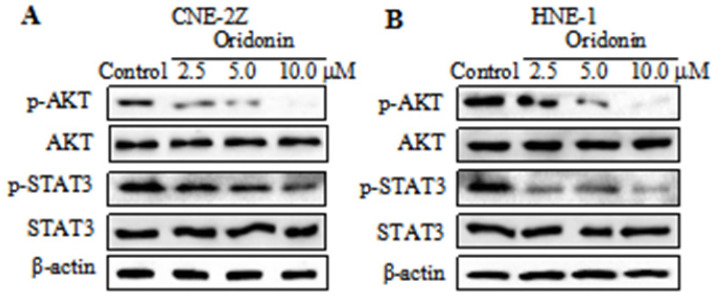
Oridonin decreased the expressions of p-AKT and p-STAT3 in nasopharyngeal carcinoma cells. Western blotting was used to examine the protein levels of phosphorylated-AKT, AKT, phosphorylated-STAT3, STAT3 and β-actin in nasopharyngeal carcinoma CNE-2Z (A) and HNE-1 (B) cells upon oridonin treatment.

Studies reported that AKT/STAT3 signaling pathway is involved with cancer metastasis 12-13. So we detected the expressions of AKT and STAT3 in nasopharyngeal carcinoma CNE-2Z cells. As depicted in Fig. 4A, a dose-dependent decrease in the phosphorylation levels of AKT and STAT3 was visualized in the CNE-2Z cell line upon oridonin treatment, while the total AKT and STAT3 exhibited no change under oridonin treatment. Furthermore, the results also revealed a concentration-dependent decrease in phosphorylated-AKT and phosphorylated-STAT3 in HNE-1 cells treated with oridonin, which was consistent with the above data (Fig. 4B). These data indicated that oridonin negatively regulated p-AKT/p-STAT3 signaling pathway.

### The anti-metastatic phenotype of oridonin is mediated by the inactivation of AKT/STAT3 signaling pathway

**Figure 5:**
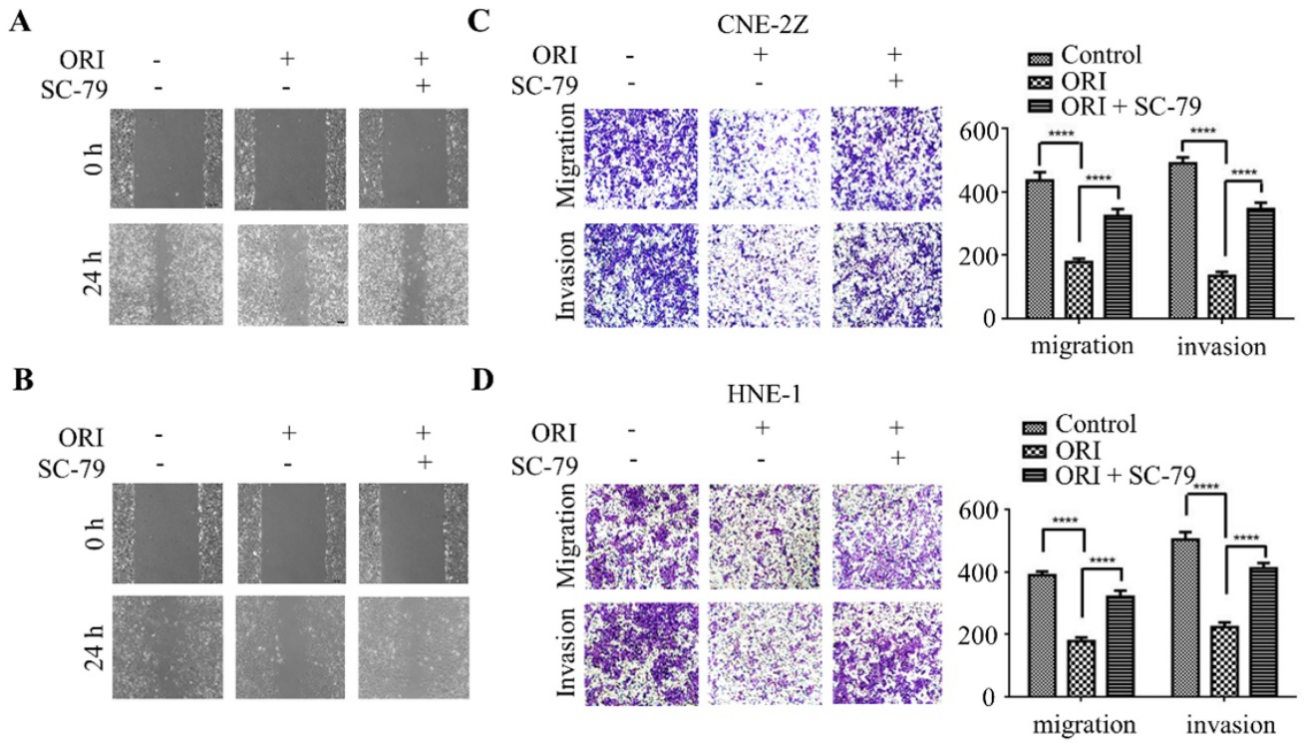
Overexpression of AKT with SC-79 attenuates the anti-metastatic phenotype of oridonin (ORI) in nasopharyngeal carcinoma cells. Wound healing assay was performed on CNE-2Z (A) and HNE-1 (B) cells following combination treatment of oridonin and SC-79. Transwell migration assay and Matrigel invasion assay were conducted on CNE-2Z (C) and HNE-1 (D) cells with the synergistic treatment of oridonin and SC-79 (magnification, ×100). Quantification of three independent experiments was shown (****P<0.0001).

**Figure 6:**
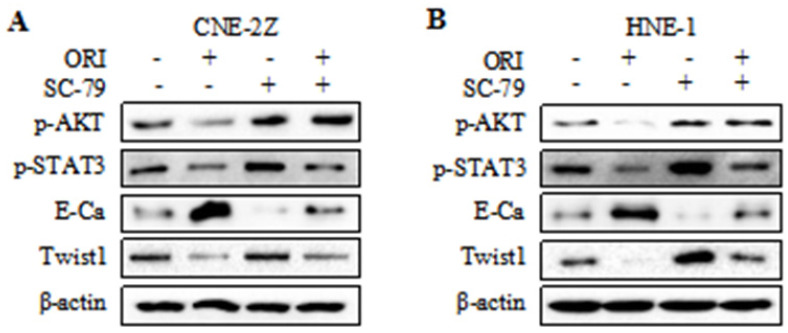
Overexpression of AKT with SC-79 partially abolished the reversal of EMT induced by oridonin (ORI) in nasopharyngeal carcinoma cells. Western blotting was used to detect the protein levels of p-AKT, p-STAT3, E-Cadherin (E-Ca), Twist1 and β-actin in CNE-2Z (A) and HNE-1 (B) cells following combination treatment of oridonin and SC-79.

To further validate whether AKT/STAT3 signaling pathway is associated with oridonin-inhibited cell migration and invasion, SC-79 (AKT activator) was used in combination with oridonin for the subsequent experiment. The results demonstrated that co-treatment with SC-79 attenuated the anti-metastatic effect of oridonin on CNE-2Z cells, as evidenced by the wound healing assay and transwell assay (Fig. 5A and 5C). And the anti-migratory and anti-invasive capability of oridonin could also be partially reversed by SC-79 in HNE-1 cell line (Fig. 5B and 5D). Moreover, oridonin-mediated upregulation of E-cadherin and downregulation of p-STAT3 and twist1 were abolished by the synergistic treatment with SC-79 to some extent in CNE-2Z and HNE-1 cells (Fig. 6A and 6B). These findings strongly supported that oridonin showed a remarkable anti-metastatic effect by the inactivation of AKT/STAT3 signaling.

## Discussion

In the past decades, the strong anti-cancer activity of oridonin attracted oncologists' and clinicians' attention 14-15. Studies showed that oridonin could induce mitochondria-dependent apoptosis in human esophageal cancer 16. And oridonin was found to exhibit a pro-apoptotic effect on osteosarcoma 17. Additionally, oridonin could inhibit the BGC823 xenograft growth in human gastric cancer in a dose-dependent manner 18. In the present study, it is the first time to our knowledge to explore the role of oridonin in metastasis in human nasopharyngeal carcinoma. And the results revealed that oridonin significantly inhibited nasopharyngeal carcinoma migration and invasion, suggesting that oridonin might be a potential therapeutic agent for nasopharyngeal carcinoma.

EMT is a complicated process including the loss of cell polarity, cell-cell adhesion and the acquisition of mesenchymal features, which allows cancer cells invading and colonizing into the distant solid organs 19. Consequently, EMT process is essential for the cancer metastasis. The hallmarks of EMT include the reduction of E-cadherin and cytokeratins, accompanied with the concomitant increase in mesenchymal indicators, such as vimentin and α-smooth muscle actin (α-SMA) 5. Nevertheless, studies showed that EMT may contribute to chemoresistance, while not to lung metastasis 20. And metastasis could occur via MET-dependent and MET-independent pathway 21. In the current study, an induced expression of E-cadherin while a decreased expression of vimentin, twist1 and a reduced mobility in nasopharyngeal carcinoma were observed upon oridonin treatment, indicating the reversal effect of oridonin on EMT in nasopharyngeal carcinoma.

EMT-mediated metastatic cascade is regulated by a variety of signaling pathways. It has been reported that sonic hedgehog signaling could facilitate tumorigenicity by activating EMT of bladder cancer 22. Previous studies also have shown that EMT could be regulated by notch signaling pathway in tumor progression 23. JAK2/STAT3 signaling was found to be involved with transforming growth factor β1-induced EMT 24. For AKT/STAT3 signaling, it participated in NFIB-mediated EMT of gastric cancer 12. However, whether AKT/STAT3 signaling pathway was involved in the oridonin-mediated MET in nasopharyngeal carcinoma has not been clarified. The current study demonstrated that oridonin combined with SC-79 could attenuate the anti-metastatic effect of oridonin on carcinoma. Additionally, the protein level of E-cadherin was down while vimentin and twist1 levels partially recovered. These findings concluded that AKT/STAT3 signaling may be a novel driver of tumor metastasis in nasopharyngeal carcinoma, thus AKT/STAT3 pathway blockage by oridonin is considered as an alternative strategy for the treatment of nasopharyngeal carcinoma.

## Conclusion

Our results revealed that oridonin inhibits nasopharyngeal carcinoma cell migration and invasion, and reverse EMT via the inactivation of AKT/STAT3 signaling pathway. It is suggested that AKT/STAT3 signaling could be identified as the potential therapeutic target of oridonin against nasopharyngeal carcinoma.
